# New species of *Phanerotomella* (Hymenoptera, Braconidae, Cheloninae) based on morphological and molecular evidence

**DOI:** 10.3897/BDJ.13.e171754

**Published:** 2025-10-01

**Authors:** Yu Fang, Jia-yue He, Cornelis van Achterberg, Jia-chen Zhu, Xue-xin Chen, Pu Tang

**Affiliations:** 1 Zhejiang Provincial Key Laboratory of Biology and Ecological Regulation of Crop Pathogens and Insects, Zhejiang University, Hangzhou, China Zhejiang Provincial Key Laboratory of Biology and Ecological Regulation of Crop Pathogens and Insects, Zhejiang University Hangzhou China; 2 Institute of Insect Sciences, College of Agriculture and Biotechnology, Zhejiang University, Hangzhou, China Institute of Insect Sciences, College of Agriculture and Biotechnology, Zhejiang University Hangzhou China; 3 State Key Lab of Rice Biology and Breeding, Zhejiang University, Hangzhou, China State Key Lab of Rice Biology and Breeding, Zhejiang University Hangzhou China; 4 Ministry of Agriculture and Rural Affairs, Key Lab of Molecular Biology of Crop Pathogens and Insects, Zhejiang University, Hangzhou, China Ministry of Agriculture and Rural Affairs, Key Lab of Molecular Biology of Crop Pathogens and Insects, Zhejiang University Hangzhou China

**Keywords:** DNA barcodes, key, new species, taxonomy

## Abstract

**Background:**

The genus *Phanerotomella* Szépligeti, 1900 (Hymenoptera, Braconidae, Cheloninae, Phanerotomini), is restricted to the Old World, with no records from the Nearctic or Neotropical Regions. Reliably species-identified COI sequences are scarce for this genus in public databases, hindering comprehensive phylogenetic and DNA-barcoding analyses.

**New information:**

This study provides 29 COI sequences for this genus. Two species delimitation approaches, combined with morphological evidence, were employed to delimit species. The findings indicated the existence of 16 species, including one new species: *P. nitidifascia* Fang, He, van Achterberg, sp. nov. and one newly-recorded species: *P. namkyensis* Sigwalt, 1978. Additionally, we provide the first available COI sequence for the Chinese endemic genus *Siniphanerotomella*.

## Introduction

*Phanerotomella* Szépligeti, 1900 is a genus currently containing about 106 species, which are recorded from all zoogeographic regions, except the Nearctic and Neotropical (He et al. 2025). Although the genus was established in 1900, the study of it can be traced back to Herrich-Schäffer (1838), who described the first species, *Chelonus bisulcatus* Herrich-Schäffer, 1838, under the genus *Chelonus* Panzer. This species was subsequently transferred to the genus *Ascogaster* by Dalla (1898) and later to *Phanerotomella* by Fahringer (1934).

Research on the genus *Phanerotomella* spans over 180 years, marked by pivotal contributions that have systematically advanced its taxonomy. Seminal work by Zettel (1989) established a global foundation, describing 51 species, including 26 new taxa. Further refining generic boundaries, van Achterberg (1990) developed a key to Western Palaearctic Cheloninae genera, clarifying the diagnostic morphology of *Phanerotomella*. Subsequently, Chen and Ji (2003) documented 25 Chinese species, proposing 12 as new. Most recently, the comprehensive revision by He et al. (2025) recognised 42 species in China, with 16 new species and eight newly recorded, synonymised five new species proposed by Chen and Ji (2003) and excluded *P. rufa* (Marshall, 1898) from the checklist due to morphological discrepancies.

*Phanerotomella* is similar to the genus of *Phanerotoma* in the shape of metasomal carapace, the presence of two transverse sutures on the metasoma and the weak lateral carina of mesoscutum, not lamelliform or protruding next to axillae. However, it can be easily identified by having vein 2-R1 of fore-wing present; vein CU1b of fore-wing absent, resulting in an open first subdiscal cell apico-posteriorly; antennal segments 26–60 and vein r of hind-wing absent.

The lack of reliably identified COI sequences for *Phanerotomella* in public repositories has significantly constrained robust phylogenetic studies and hindered the development of a DNA barcoding for this genus. To address this gap, we obtained 29 *Phanerotomella* COI sequences and one *Siniphanerotomella* COI sequence. Two species delimitation approaches, along with morphological evidence, were applied to identify species and investigate the intra- and interspecific variation. Our results revealed one new species, *Phanerotomella nitidifascia* Fang, He, van Achterberg, sp. nov. and one newly-recorded species from China, *P. namkyensis* Sigwalt, 1978. The new species is comprehensively described; illustrations and diagnostic notes are provided for both taxa newly reported from China.

## Materials and methods

### Taxon sampling

The specimens examined in this study were collected through the sweeping net and Malaise trap methods. All the specimens examined in this study are deposited in the Parasitic Hymenoptera Collection of Zhejiang University, Hangzhou, China (**ZJUH**).

### Specimen examination

The morphological terminology and measurements of body parts follow van Achterberg (van Achterberg 1988, van Achterberg 1993) and He et al. (2000). The detailed morphological examinations and dimensional assessments were performed by utilising a Nikon stereoscopic microscope (SMZ800N), while the illustrations were produced with the aid of a digital microscope (KEYENCE VHX-7000; Osaka, Japan). The photos were partly enhanced and laid out on a plate using Adobe Photoshop 2023. The following abbreviations of morphological terms are used: **OOL** = shortest distance from a posterior ocellus to nearest eye margin; **OD** = maximum diameter of posterior ocellus; **POL** = minimum width between posterior ocelli.

### DNA extraction, PCR amplification and sequencing

All DNA in this study was extracted using the QIAamp DNA Mini Kit (Qiagen, Hilden, Germany) and following a non-destructive protocol (Cruaud et al. 2019). Sequence data for these new species, as well as for previously described species included in the analyses, are provided in Table S1. Amplification of the approximately 658 bp fragment of COI barcode region (Hebert et al. 2003) was carried out using the primers LCO1490 and HCO2198 (Folmer et al. 1994). PCR amplifications were performed using KOD One™ PCR Master Mix and the PCR was run with the following setup: initial denaturation at 98℃ for 5 min and a five-cycle preamplification (30 s at 98℃, 40 s at 45℃ and 1 min at 72℃), followed by 35 cycles of 30 s at 98℃, 40 s at 55℃ and 1 min at 72℃ and a final extension of 5 min at 72℃.

### Data analyses

Sequencing of the final product was performed in both forward and reverse directions and edited using Geneious Prime 2024.0.5. In addition, 28 sequences of congeneric species were downloaded from BOLD Systems v.3 on 28 August 2024 (Suppl. material 1). All the sequences were translated into amino acids in Geneious Prime 2024.0.5 to identify any stop codons and then aligned using the MAFFT v.7.505 (Katoh and Standley 2013, Nakamura et al. 2018). The final alignment had a length of 683 bp and included undefined nucleotides (N) for some sequences (Suppl. material 3).

Sequence divergences for intraspecific and interspecific pairwise genetic distances were computed, based on the Kimura-2parameter (K2P) model in MEGA-X (Kumar et al. 2018) (Table S2). Maximum-Likelihood (ML) analyses were performed using IQ-TREE v. 2.1.3 (Minh et al. 2020) and the best-fitting substitution model was identified using Model Finder implemented in IQ-TREE (MFP). tvBOT (https://www.chiplot.online/tvbot.html) was utilised to visualise and illustrate the inferred phylogenetic trees (Xie et al. 2023).

Two different methods were used for species delimitation: the distance-based method Automatic Barcode Gap Discovery (ABGD) and the tree-based method Poisson Tree Process (PTP) (Zhang et al. 2013). The ABGD analysis was used for species delimitation, automatically partitioning sequences into candidate species, based on the barcode gap (difference between intra- and inter-specific variation) without needing a priori thresholds (Puillandre et al. 2012). The ABGD analysis was conducted via a windows executable (https://itaxotools.org/abgd.zip), using the K2P model to classify species based on genetic distances. The relative gap width (X) was set to 1.0 and the remaining parameters were set to default. The bPTP method was performed online (https://species.h-its.org/ptp/), with unrooted selected for tree type, 100,000 specified for NO. MCMC generations and default parameters used for the rest.

## Taxon treatments

### Phanerotomella namkyensis

Sigwalt, 1978

E8030E25-BFF5-5BDD-868C-C8C6D1A36008

PX260884

Phanerotomella namkyensis Sigwalt, 1978 - *Sigwalt (1878)*: 719; Zettel (1989): 54; Long and Belokobylskij (2004): 387.

#### Materials

- **Type status:** Other material. **Occurrence:** catalogNumber: ZJUH No. 202401002; recordedBy: Jiangli Tan; sex: female; lifeStage: adult; occurrenceID: 500A784D-DE18-551D-A170-B4C4DBCA5FA8; **Taxon:** scientificName: *Phanerotomella namkyensis* Sigwalt, 1978; **Location:** country: China; stateProvince: Hainan; locality: Yingge Ridge; **Event:** verbatimEventDate: 30 April 2010; **Record Level:** institutionCode: ZJUH; basisOfRecord: PreservedSpecimen

#### Diagnosis

Carapace dark brown, except the first and second tergites yellow medially; metasoma narrowing to apex and widest at basal half; lateral border of metasomal carapace curved; temple yellow and without two brown spots behind eye; eye in lateral view 1.3 times wider than maximum width of temple; scapus and pedicellus in lateral view ivory and distinctly contrasting with dark brown flagellum; antenna of ♀ slender, third, fourth, tenth and fifteenth segments 3.4, 3.1, 2.2 and 1.6× longer than wide in lateral view, respectively; vein m-cu of fore-wing antefurcal; hind femur 3.3 times longer than wide.

#### Distribution

**Figure 1.**
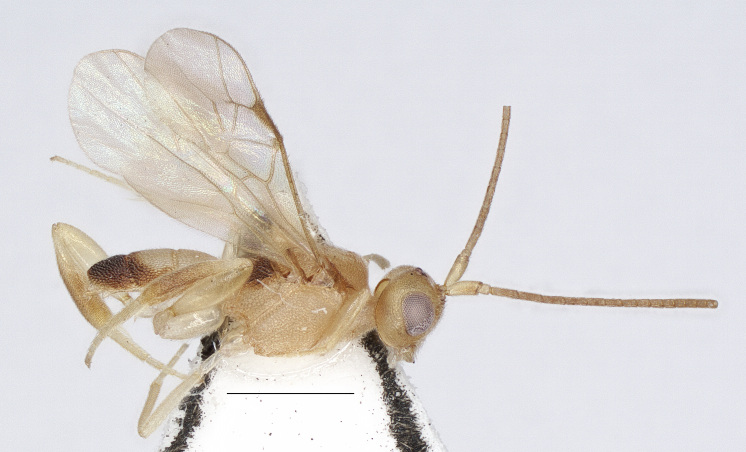
*Phanerotomella namkyensis* Sigwalt, 1978, Hainan, ♀, habitus, lateral aspect. Scale bar: 1.0 mm.

**Figure 2.**
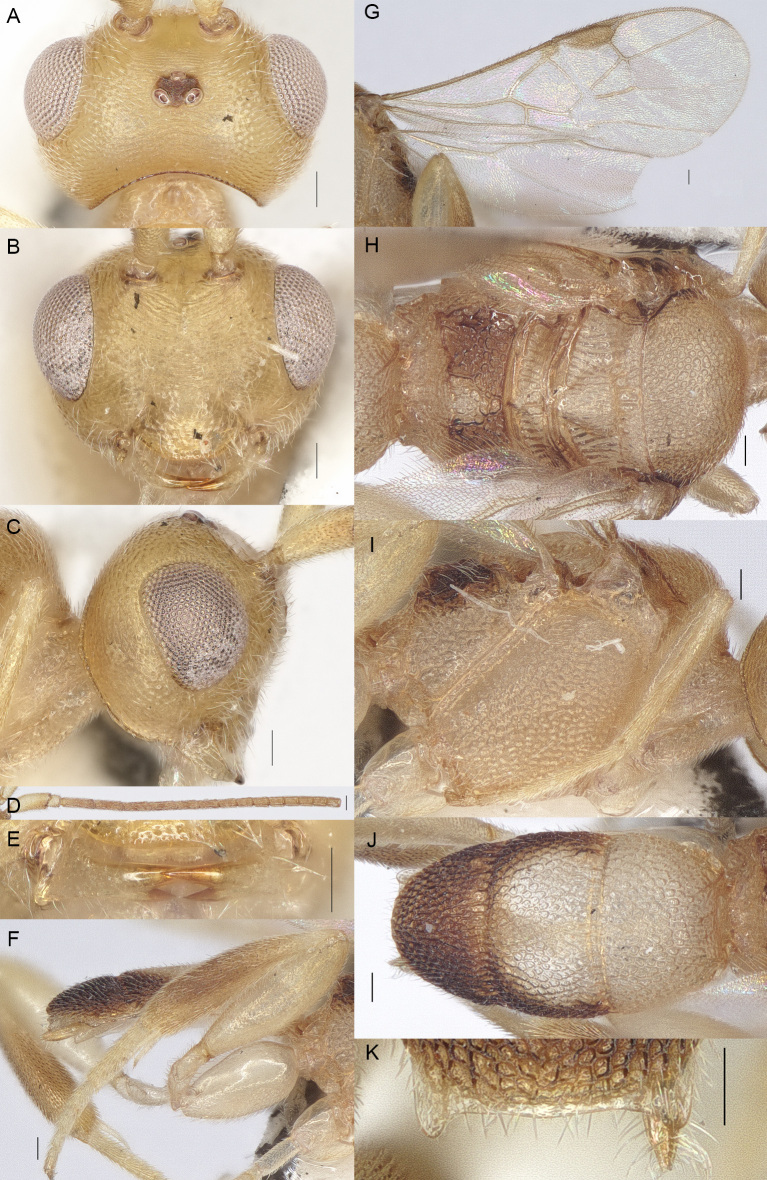
*Phanerotomella namkyensis* Sigwalt, 1978, Hainan, ♀. **A** Head dorsal; **B** Head anterior; **C** Head lateral; **D** Antenna lateral; **E** Mandible ventral; **F** Hind leg; **G** Fore-wing and hind-wing; **H** Mesosoma dorsal; **I** Mesosoma lateral; **J** Metasoma dorsal; **K** Lamella posterior. Scale bars: 0.1 mm.

China (Hainan) (Figs 1, 2), Vietnam.

#### Remarks

*P. namkyensis* Sigwalt, 1978 is similar to *P. hawaiiensis* (Ashmead, 1901) due to having similar colouration of the body and hind femur. However, *P. namkyensis* Sigwalt, 1978 can be distinguished from *P. hawaiiensis* (Ashmead, 1901) by metasoma narrowing to apex and widest at basal half (metasoma symmetrically-shaped and position of maximum width in middle in the latter), temple without two brown spots behind eye (with two brown spots behind eye in the latter), antenna of ♀ comparatively slender (widened medially in the latter), scapus and pedicellus in lateral view ivory and distinctly contrasting with dark brown flagellum (scapus and pedicellus in lateral view dark brown and no contrasting with dark brown flagellum in the latter).

### Phanerotomella nitidifascia

Fang, He & van Achterberg sp. nov.

75DFA910-077D-54A5-A214-03AF127A41AE

C93BE0E4-8749-438B-9CB2-227C39D979BE

PX260888

PX260906

#### Materials

- **Type status:** Holotype. **Occurrence:** catalogNumber: ZJUH No. 202401006; recordedBy: Manman Wang leg.; sex: female; lifeStage: adult; occurrenceID: 559FD881-28B5-5405-B74A-F9642AC15741; **Location:** country: China; stateProvince: Yunnan; county: Yingjiang; **Event:** verbatimEventDate: 19 May 2009; **Record Level:** institutionCode: ZJUH
- **Type status:** Paratype. **Occurrence:** catalogNumber: ZJUH No. 202401036; recordedBy: Jie Zeng leg.; sex: male; lifeStage: adult; occurrenceID: 5F52E01C-25DF-514B-ABC4-DFC5CE9B3333; **Location:** country: China; stateProvince: Yunnan; county: Yingjiang; **Event:** eventDate: 21 May 2009; **Record Level:** institutionCode: ZJUH; basisOfRecord: PreservedSpecimen

#### Description

**Figure 3.**
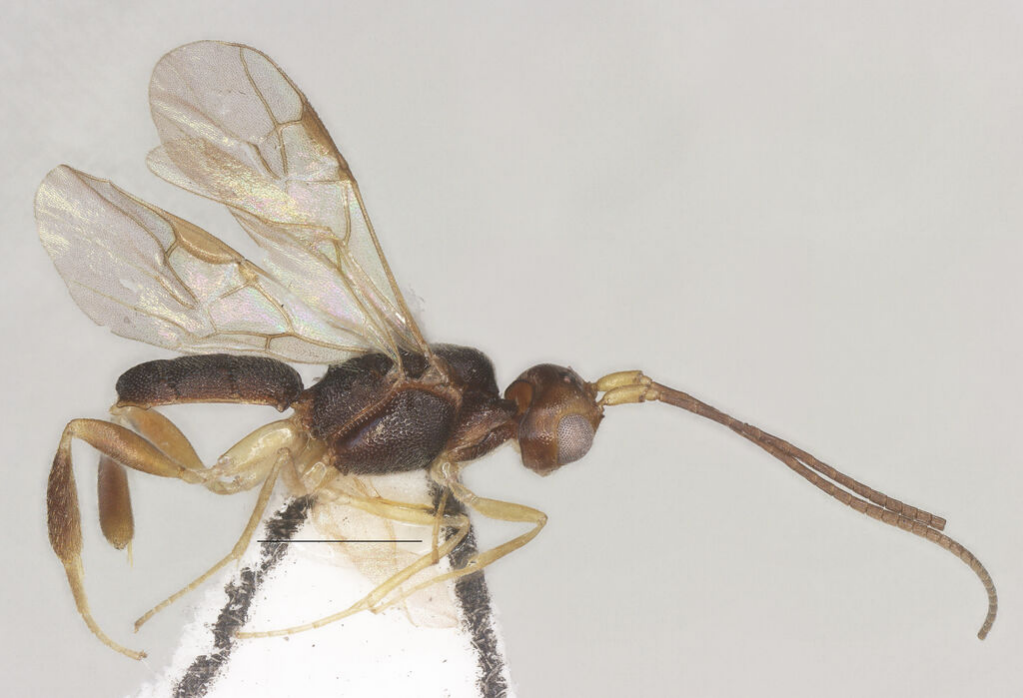
*Phanerotomella nitidifascia* sp. nov., holotype, ♀, habitus, lateral aspect. Scale bar: 1.0 mm.

Holotype. ♀ (Fig. 3). Length of body 2.8 mm, fore-wing 2.5 mm.

##### Head

**Figure 4.**
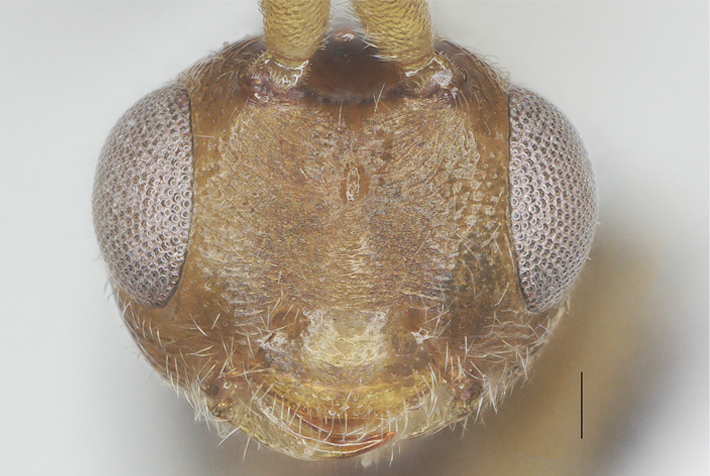
*Phanerotomella nitidifascia* sp. nov., holotype, ♀. Head anterior. Scale bar: 0.1 mm.

**Figure 5.**
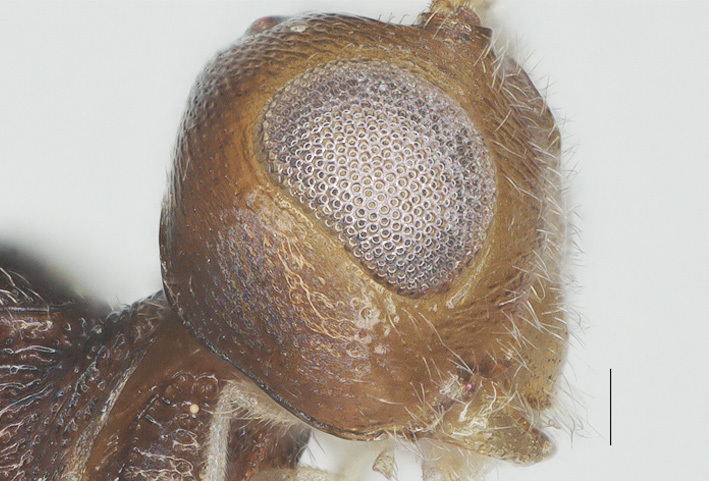
*Phanerotomella nitidifascia* sp. nov., holotype, ♀. Head lateral. Scale bar: 0.1 mm.

**Figure 6.**
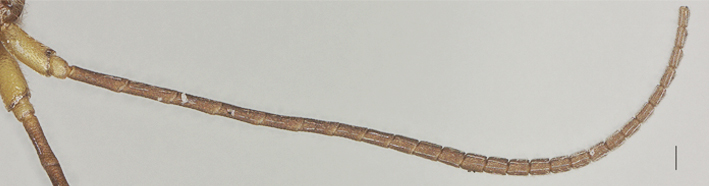
*Phanerotomella nitidifascia* sp. nov., holotype, ♀. Antenna lateral. Scale bar: 0.1 mm.

**Figure 7.**
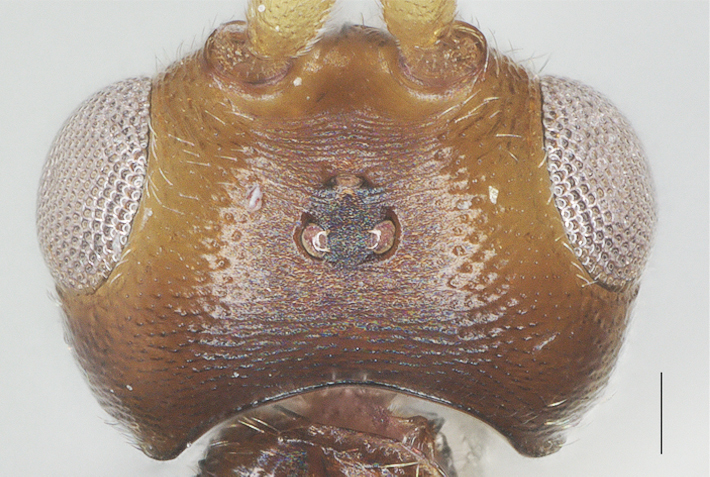
*Phanerotomella nitidifascia* sp. nov., holotype, ♀. Head dorsal. Scale bar: 0.1 mm.

**Figure 8.**
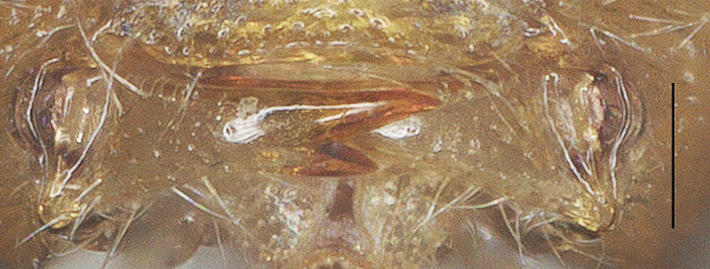
*Phanerotomella nitidifascia* sp. nov., holotype, ♀. Mandible ventral. Scale bar: 0.1 mm.

Width 1.3× median length in anterior view (Fig. 4) and part of head above eye in lateral view 0.3× height of eye (Fig. 5); antenna with 28 segments, but apical segments missing, slightly widened and shortened medially, gradually narrowed apically, subapical segments non-moniliform and longer than wide, third, fourth, tenth and fifteenth segments 3.8, 3.5, 1.7 and 1.3× longer than wide in lateral view, respectively (Fig. 6); area of stemmaticum transversely striate; OOL: OD: POL = 4: 1: 1; length of eye 1.5× temple in dorsal view (Fig. 7); frons transversely rugulose and with median carina; vertex rugulose-punctate with short setae; temple superficially rugulose and somewhat shiny; face rugulose and with distinct median ridge, dorsally connecting to median carina; clypeus smooth and shiny, except for finely punctation, truncate medio-ventrally; eye width in lateral view 1.3× maximum width of temple (Fig. 5), eye height in anterior view 0.7× minimum width of face (Fig. 4); malar space rugulose and 1.2× as basal width of mandible; mandible rather slender, lower tooth of mandible 0.5× as long as apical tooth (Fig. 8); face width equal to height of face and clypeus together.

##### Mesosoma

**Figure 9.**
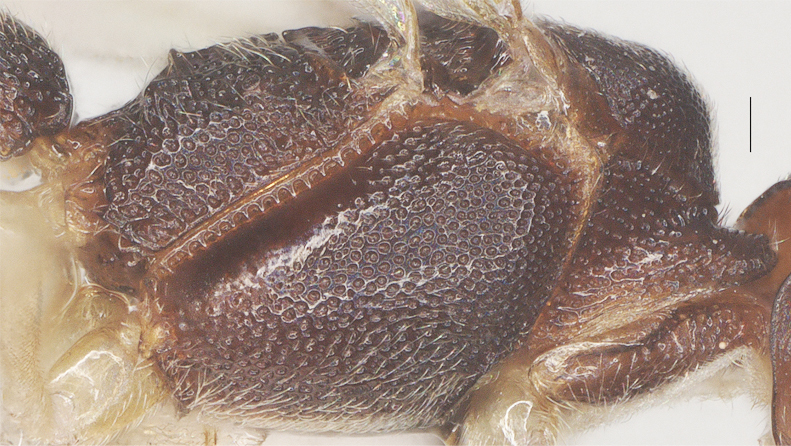
*Phanerotomella nitidifascia* sp. nov., holotype, ♀. Mesosoma lateral. Scale bar: 0.1 mm.

**Figure 10.**
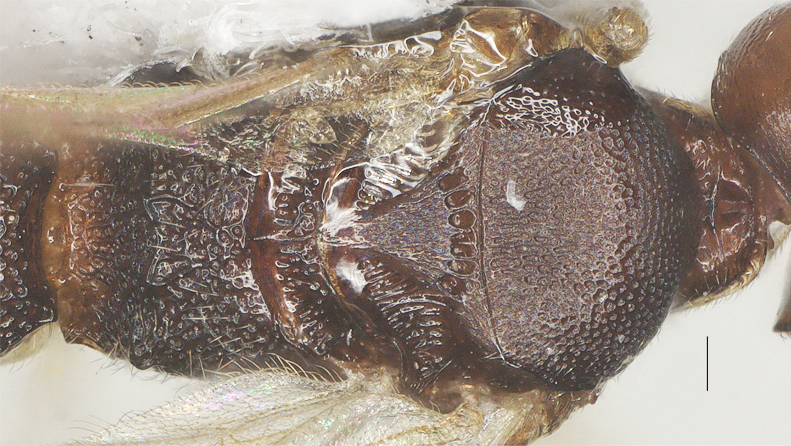
*Phanerotomella nitidifascia* sp. nov., holotype, ♀. Mesosoma dorsal. Scale bar: 0.1 mm.

Length 1.6× its width in lateral view; side of pronotum mainly superficially and finely punctate, except anteriorly rugulose; mesoscutum densely reticulate; notauli absent; scutellar sulcus with six short crenulae; scutellum rugulose; mesopleuron punctate-reticulate and with smooth and shiny area posteriorly, precoxal sulcus absent; propodeum reticulate, without median carina, with irregular transverse carina connected to four weak and blunt lateral tubercles (Fig. 9, Fig. 10).

##### Wings

**Figure 11.**
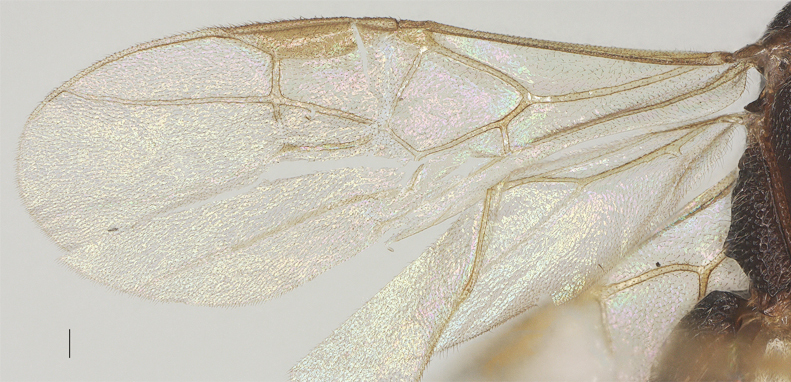
*Phanerotomella nitidifascia* sp. nov., holotype, ♀. Fore-wing and hind-wing. Scale bar: 0.1 mm.

Fore-wing 2.6× longer than its maximum width; second submarginal cell not petiolate; vein m-cu weakly antefurcal; vein r and 1-SR+M straight; vein SR1 slightly curved; length of 1-R1 1.0× pterostigma; vein r issued far beyond middle of pterostigma, 0.9× vein r-m; r:2-SR:SR1 = 13: 35: 70; vein 1-CU1 0.4× as long as vein 2-CU1. Hind-wing: M+CU:1-M:1r-m = 23: 31: 14 (Fig. 11).

##### Legs

**Figure 12.**
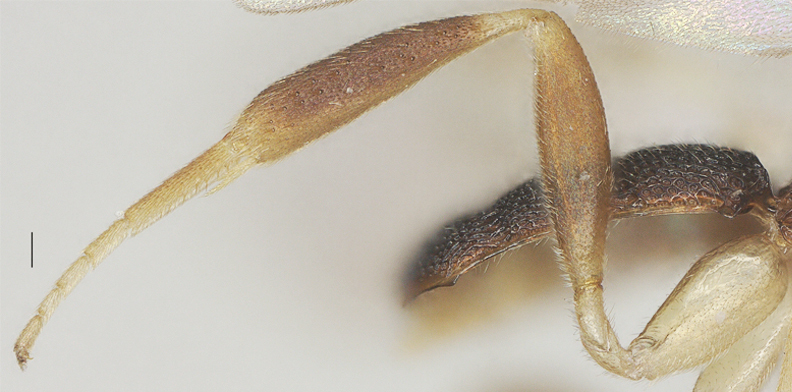
*Phanerotomella nitidifascia* sp. nov., holotype, ♀. Hind leg. Scale bar: 0.1 mm.

Hind femur 3.8× as long as wide; longest spur of hind tibia 0.5× its basitarsus; hind leg smooth and shiny, except fine and superficial punctation; middle tibia without ivory blister (Fig. 12).

##### Metasoma

**Figure 13.**
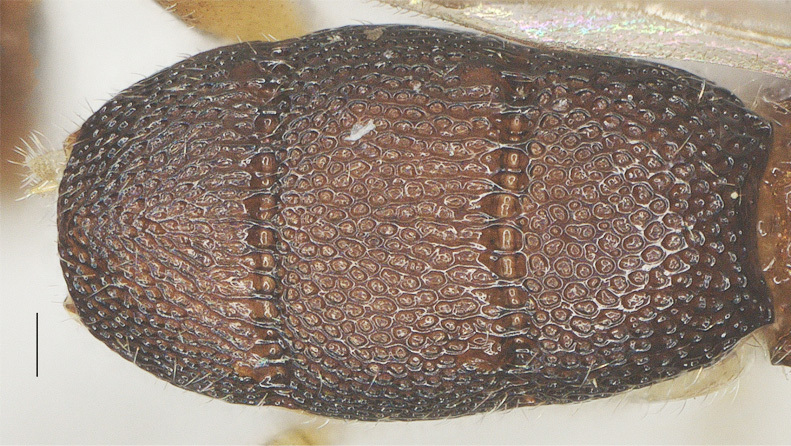
*Phanerotomella nitidifascia* sp. nov., holotype, ♀. Metasoma dorsal. Scale bar: 0.1 mm.

**Figure 14.**
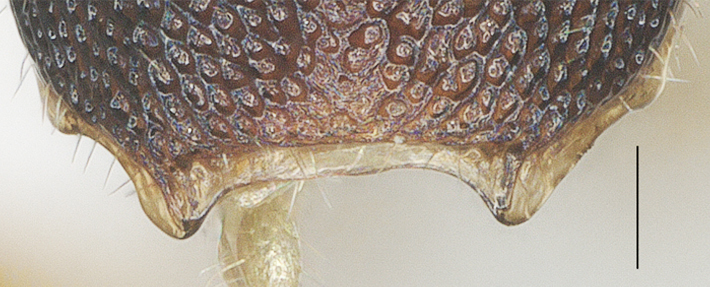
*Phanerotomella nitidifascia* sp. nov., holotype, ♀. Lamella posterior. Scale bar: 0.1 mm.

Oval in dorsal view (Fig. 13), carapace 1.8× as long as wide and as long as mesosoma; first to third tergites densely reticulate-rugose; third tergite 0.9× as long as second tergite, medial length of third tergite 0.6× its maximum width; lamella of third tergite not protruding medio-apically and with pair of distinct and blunt tubercles latero-apically (Fig. 14).

##### Color

Black; head yellowish-brown, but black around occipital carina; antenna dark brown, except for yellow scapus and pedicellus; mandible yellowish and with reddish teeth; fore- and middle legs yellow; hind leg dark brown, except coxa, trochanter and base of tibia ivory, tarsus yellowish-brown; pterostigma and parastigma brown, wing veins pale brown.

##### Male

Very similar to female, but antenna slender medially and second submarginal cell weakly petiolate. Antenna with 33 segments.

#### Diagnosis

Mesopleuron punctate-reticulate and with distinctly smooth area posteriorly; lamella of third metasomal tergite more protruding latero-apically; temple superficially rugulose and somewhat shiny; posterior ocellus comparatively small; vein m-cu of fore-wing antefurcal; scapus and pedicellus in lateral view yellow and distinctly contrasting with dark brown flagellum.

*Phanerotomella nitidifascia* sp. nov. is similar to *P. exilicornis* He et al., 2025, because of similar colouration of the body, hind femur and antenna and vein m-cu antefurcal. However, *P. nitidifascia* sp. nov. can easily be distinguished from *P. exilicornis* by having mesopleuron with a smooth and shiny area posteriorly (without a smooth area in the latter), lamella of third metasomal tergite distinctly protruding latero-apically (less protruding latero-apically in the latter), temple superficially rugulose and somewhat shiny (distinctly rugulose and matt in the latter) and posterior ocellus comparatively small (posterior ocellus large and round in the latter).

#### Etymology

Named after its mesopleuron with distinctly smooth and shiny area posteriorly; “nitidus” is Latin for smooth and shiny; “fascia” is Latin for strip.

#### Distribution

China (Yunnan)

#### Biology

Unknown.

## Analysis

**Figure 15.**
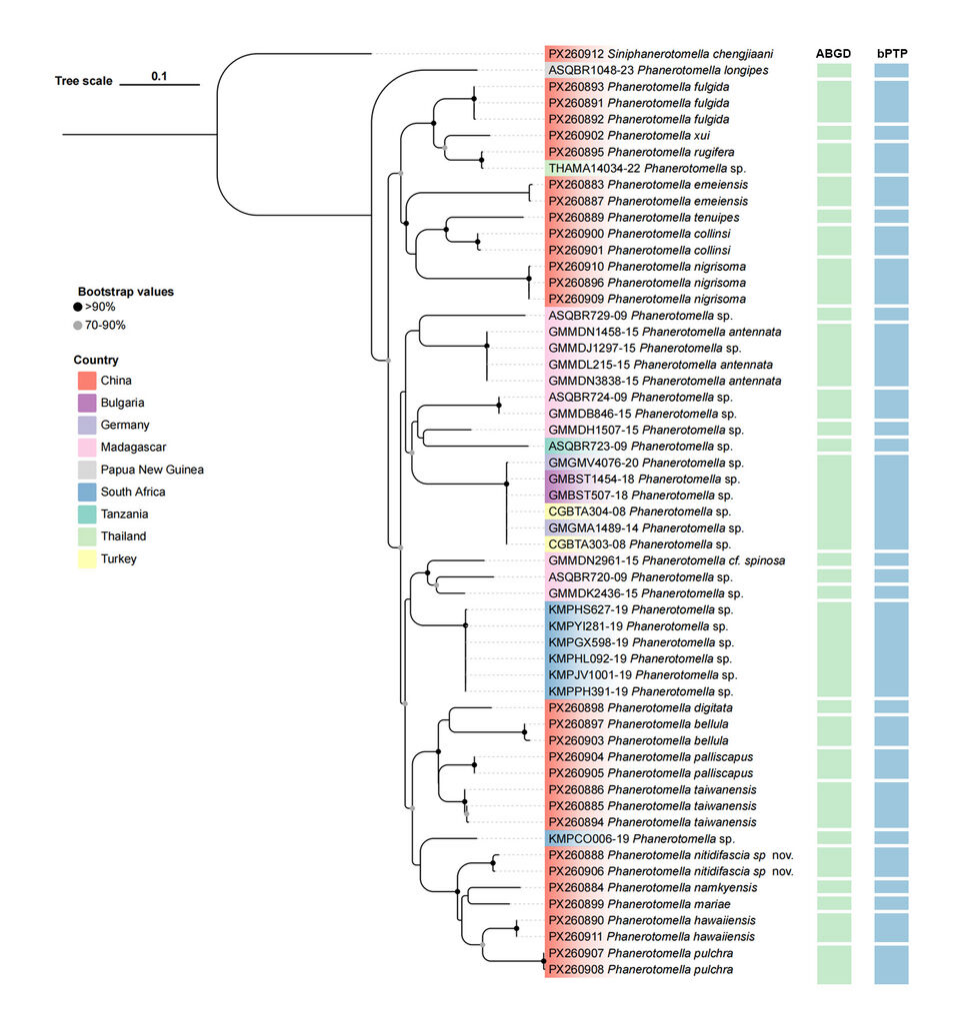
ML phylogenetic tree, based on 58 COI sequences highlighting the results of two delimitation analyses in *Phanerotomella*. The results of delimitation analyses are displayed with the vertical bars corresponding to putative species (MOTUs) inferred by ABGD and bPTP methods.

The COI sequence of *Phanerotomella* was successfully amplified from 29 specimens, deposited in GenBank (accession numbers in Suppl. material 1). Species delimitation results from the two approaches are summarised in Fig. 15. Both species delimitation methods consistently delineated all sequences into 28 MOTUs, and combined with morphological evidence, we successfully identified 16 species, comprising one new species: *P. nitidifascia* sp. nov., one newly-recorded species: *P. namkyensis* and 14 previously known species: *P. bellula*, *P. collinsi*, *P. digitata*, *P. emeiensis*, *P. fulgida*, *P. hawaiiensis*, *P. mariae*, *P. nigrisoma*, *P. palliscapus*, *P. pulchra*, *P. rugifera*, *P. taiwanensis*, *P. tenuipes* and *P. xui*.

Furthermore, we also estimated pairwise distances using the P-distance model with pairwise deletion and found that interspecific distances ranged from 0.0577 to 0.2198 (Table 1; Suppl. material 2). The analysis of pairwise genetic distances provided strong molecular support for the species delimitation by both morphological examination and MOTU clustering. Notably, based on genetic distances, *P. nitidifascia* sp. nov. is relatively close to *P. mariae* (0.0774) and *P. namkyensis* (0.0801). However, it can be easily distinguished from the former species by antenna of ♀ slender, slightly widened and shortened medially, gradually narrowed apically; mesopleuron punctate-reticulate and shiny and with a smooth and shiny area posteriorly; lamella of third metasomal tergite not protruding medio-apically. *P. nitidifascia* sp. nov. can be easily distinguished from *P. namkyensis* by mesopleuron punctate-reticulate and shiny and with a smooth and shiny area posteriorly; metasoma narrowing to base and widest at apical half; body black. These congruent results from both molecular and morphological evidence collectively affirm the validity of *P. nitidifascia* sp. nov. as a distinct species within the genus.

## Supplementary Material

XML Treatment for Phanerotomella namkyensis

XML Treatment for Phanerotomella nitidifascia

DC62DB27-2DC7-5D52-AB8E-5EA7239F8470 10.3897/BDJ.13.e171754.suppl1 Supplementary material 1 Information of terminal taxa of Phanerotomella molecular analysis Data type information File: oo_1412095.xlsx https://binary.pensoft.net/file/1412095 Yu Fang, Jiayue He, Cornelis van Achterberg, Jiachen Zhu, Xuexin Chen and Pu Tang

B4C4A284-933C-5BBC-9E79-74A44974ED40 10.3897/BDJ.13.e171754.suppl2 Supplementary material 2 MEGA-K2p-distance Data type genetics distance File: oo_1421719.xlsx https://binary.pensoft.net/file/1421719 Yu Fang, Jiayue He, Cornelis van Achterberg, Jiachen Zhu, Xuexin Chen and Pu Tang

485E50FB-4B48-5D36-AC20-C82A35FAFA84 10.3897/BDJ.13.e171754.suppl3 Supplementary material 3 Phanerotomella.align.fasta Data type fasta File: oo_1412180.fasta https://binary.pensoft.net/file/1412180 Yu Fang, Jiayue He, Cornelis van Achterberg, Jiachen Zhu Xuexin Chen and Pu Tang

## Figures and Tables

**Table 1.** Species interspecific distances range.

	MIN	MAX
*P. bellula*	0.0914	0.1835
*P. collinsi*	0.0952	0.2029
*P. digitata*	0.0750	0.1727
*P. emeiensis*	0.1342	0.1956
*P. fulgida*	0.0796	0.1720
*P. hawaiiensis*	0.0841	0.1849
*P. mariae*	0.0774	0.1892
*P. namkyensis*	0.0801	0.1836
*P. nitidifascia* sp. nov.	0.0773	0.1800
*P. nigrisoma*	0.1430	0.1911
*P. palliscapus*	0.0577	0.1691
*P. pulchra*	0.0848	0.2036
*P. rugifera*	0.0735	0.1780
*P. taiwanensis*	0.0577	0.1570
*P. tenuipes*	0.0952	0.2198
*P. xui*	0.0735	0.1982
GMBST1454-18 *Phanerotomella* sp.	0.1153	0.2046
ASQBR729-09 *Phanerotomella* sp.	0.1302	0.2198
ASQBR720-09 *Phanerotomella* sp.	0.0818	0.1911
ASQBR724-09 *Phanerotomella* sp.	0.1144	0.1914
GMMDH1507-15 *Phanerotomella* sp.	0.0997	0.1688
GMMDN1458-15 *P. antennata*	0.0988	0.1808
GMMDK2436-15 *Phanerotomella* sp.	0.0818	0.1696
GMMDN2961-15 Phanerotomella cf. spinosa	0.0917	0.1843
ASQBR1048-23 *Phanerotomella longipes*	0.1442	0.1883
KMPCO006-19 *Phanerotomella* sp.	0.9338	0.1971
KMPGX598-19 *Phanerotomella* sp.	0.0941	0.1714
ASQBR723-09 *Phanerotomella* sp.	0.1053	0.1911
